# Supplementary material for: A Simulation-Based Clinical Nursing Education Framework for a Low-Resource Setting: A Multimethod Study
Source: Healthcare (Basel). 2022 Aug 28;10(9):1639. doi: 10.3390/healthcare10091639 (PMC9498921; doi:10.3390/healthcare10091639)
Supplement: Supplementary file 1 [file healthcare-10-01639-s001.zip › healthcare-1847340-supplementary.pdf]

Table S1. Synthesis of findings and design characteristics from scoping review, focus group discussions, and individual interviews

CONSTRUCTS - CONECPTUAL FRAMEWORK	Scoping Review	Focus Group Discussions (FGD)	Individual Interviews	Synthesized Findings
**Context**	Establishing the physical environment of occurrence of the simulation activity whether in the skills laboratory or in the clinical setting.<br><br>Establishing whether the simulation is to be implemented within a high or a low resource setting.<br><br>Identifying the overall purpose of the simulation, whether for instruction or evaluation.<br><br>Identifying the physical measures to put in place to prevent physical harm to simulated participants.<br><br>Identifying participant expectations and the primary goal of the simulation	Students felt the skills laboratory was not conducive to support student learning.	.	The "context" is broadened to include the identification of physical measures such as the provision of adequate skills laboratory space and chairs to ensure a conducive learning environment for students.
**Background**	The background includes the needed resources (space, equipment, and personnel) and their allocation for the simulation.<br><br>Ensuring alignment between the curriculum content and the simulation activities.<br>Curriculum integration	Lack of the needed resources such as adequate skills laboratory space and equipment (in both the skills laboratory and the clinical setting), and inability of nurse educators to adopt contemporary practical skills teaching strategies.	Lack of the needed resources such as adequate skills laboratory space, equipment (in both the skills laboratory and the clinical setting), and lack of opportunity for enhancing the capacity and professional growth and development of nurse educators especially in practical skills teaching.<br><br>Lack of adequate alignment between the curriculum content and practical skills teaching strategies.<br>Lack of integration of practical skills teaching strategies in the curriculum.	The construct "planning" was introduced to broadly address the specific challenges of low-resource setting as identified in the focus group discussions and individual interviews. The adoption of simulation-based nursing education (SBNE) as a teaching strategy requires thorough planning. Considering that simulation-based nursing education is a new concept in low-resource settings, effective planning is needed to ensure a successful design and implementation of the concept. As part of the planning, there is the need for an extensive needs assessment, pre-training of nurse

CONSTRUCTS - CONECPTUAL FRAMEWORK	Scoping Review	Focus Group Discussions (FGD)	Individual Interviews	Synthesized Findings
	Structuring the simulation design with a learning theory or framework		No context specific framework to guide the design, implementation, and evaluation of simulation in low-resource settings.	educators to act as simulation facilitators, and adequate integration of simulation into the curriculum. The development of this framework will also address the need for a context-specific framework to guide the design. Implementation, and evaluation of simulation in low-resource settings.
**Simulation Design**	Formulating well spelt out, concise, and measurable learning objectives.<br>Developing simulation scenarios and ensuring their problem-solving complexity are guided by the learning objectives.	Unavailability of learning objectives to guide the teaching of practical skills.<br>Unavailability of learning objectives to guide the teaching of practical skills.	Lack of competency-based curriculum with vague learning objectives.<br>Lack of expertise and training of nurse educators on contemporary practical skills teaching strategies such as simulation-based clinical nursing education.	The contextual elements identified through the needs assessment are expected to direct the construction or formulation of the broad simulation goals, which in turn will influence the design of the simulation learning objectives. The need for the inclusion of simulation modality and the use of low-fidelity simulation modalities as components of the simulation-based clinical nursing education (SBCNE) framework is crucial to specifically tailor simulation to the needs and resources of low-resource settings, especially, using students to act out the role play, or using an orange fruit to demonstrate the administration of intramuscular injection.<br>The inclusion of videography and structured debriefing in the graphical illustration of the framework is essential to buttress the relevance of those components in the simulation activity.
	Keeping participants fully immersed by sustaining realism (fidelity) of the simulation activity.		Lack of expertise and training of nurse educators on contemporary practical skills teaching strategies such as simulation-based clinical nursing education.	
	Ensuring all scenarios begin with a pre-briefing and orientation session to set the stage for an effective simulation experience.	Role play and problem-based learning strategies were rarely used for practical skills teaching.	Lack of expertise and training of nurse educators on contemporary practical skills teaching strategies such as simulation-based clinical nursing education.	
	Ensuring dress rehearsal for all simulated participants and participants who are to act out the role play.	Role play and problem-based learning strategies were rarely used for practical skills teaching.	Lack of expertise and training of nurse educators on contemporary practical skills teaching strategies such as simulation-based clinical nursing education.	
	Choosing the most appropriate simulation modality (high fidelity manikins, low fidelity manikins, role play and standardised patients to match with the learning objectives and simulation scenario.	Simulation-based nursing education was not identified as a concept used for practical skills teaching.	Lack of expertise and training of nurse educators on contemporary practical skills teaching strategies such as simulation-based clinical nursing education.	
	Ensuring a structured debriefing immediately after the simulation experience.	Simulation-based nursing education was not identified as a concept used for practical skills teaching.	Lack of expertise and training of nurse educators on contemporary practical skills	

CONSTRUCTS - CONECPTUAL FRAMEWORK	Scoping Review	Focus Group Discussions (FGD)	Individual Interviews	Synthesized Findings
			teaching strategies such as simulation-based clinical nursing education.	
**Simulation Experience**	Establishing an environment characterised by mutual trust between the facilitator and participants, experiential, interactive, collaborative, and learner centred.	The attitude of nurse educators instilled fear in students derailing mutual trust. The learning activity is characterised by more teacher-centred teaching and learning strategies such as the use of lectures.	Disinterest and commitment of students in the learning of practical skills. The learning activity is characterised by more teacher-centred teaching and learning strategies such as the use of lectures.	Central to the success of the simulation experience is the need for the establishment of an effective community of learning. The introduction of simulation community of learning as a construct of the SBCNE framework is essential to demonstrate the dynamic interplay between facilitators who are expected to be knowledgeable in simulation and debriefing, participants with the right attitude towards learning, as well as the use of effective teaching and learning strategies, all of which must exist within an environment of effective teamwork and mutual trust. The "simulation community of learning construct", therefore, needs to broadly includes; the simulation facilitators, participants, other members of the simulation community of learning (clinicians, other participants, other nurse educators, and regulatory bodies), immersive teaching and learning strategies, and team work. The inclusion of other members (clinicians, other participants, other nurse educators, and regulatory bodies) as a component of the simulation community of learning is to facilitate the introduction and sustainability of simulation in low-resource settings. The inclusion of clinicians as part of other members of the simulation community of learning is to ensure uniformity in performing nursing procedures in practical skills teaching in order to reduce the theory-practice gap as identified in the empirical studies. This is
**Facilitator and Educational Strategies**	The facilitator is described as a person with the responsibility of providing support for students during the simulation activity.	The nurse educator is seen as a person with the responsibility of providing support and guidance for students during practical skills teaching.	The nurse educator is a person with the responsibility of providing support and guidance for students during practical skills teaching.	
	Selected demographics such as age, years of experience, and clinical expertise are believed to be related to the facilitator role.	The nurse educator must be experienced.		
	The facilitator must be knowledgeable and clinically competent.	The nurse educator must be knowledgeable and competent in clinical skills.	Nurse educators lack the necessary knowledge and contemporary skills in the teaching of practical skills and in facilitating the development of clinical competence.	
	The facilitator guides the simulation experience by altering the educational strategies such as planned progression and timing of the simulation activities and providing feedback – cues, and debriefing at the end of the simulation experience.		There is the need for an effective training of nurse educators to be equipped with knowledge and skills in the teaching of facilitating clinical skills and in facilitating the development of clinical competence.	
**Participant**	Participant denotes a person who participate in simulation activities to gain knowledge and master skills in readiness to assume professional role.	Participant denotes a person (student) who participate in the practical skills teaching and learning to gain knowledge and master skills in readiness to assume professional role. Large student numbers was identified as a challenge.	Participant denotes a person (student) who participate in the practical skills teaching and learning to gain knowledge and master skills in readiness to assume professional role. Large student numbers was identified as a challenge.	

CONSTRUCTS - CONECPTUAL FRAMEWORK	Scoping Review	Focus Group Discussions (FGD)	Individual Interviews	Synthesized Findings
	The participant must possess both innate (age, gender, level of anxiety, and self-confidence) and modifiable [preparedness for the simulation] attributes.	The participant must show interest and commitment in the learning process. The lack of interest and commitment of participants in the teaching and learning process affects the attainment of the needed practical skills.	Lack of seriousness of participants to engage in the teaching and learning of practical skills.	expected to reduce the stress on nurse educators and participants and ensure sustained learning.<br>To address the issue of large student numbers, as revealed by the empirical studies, the concept of station teaching will be incorporated into the framework as a component of immersive teaching and learning strategies.
**Outcomes**	Simulation outcomes are focused on participant, patient, and system.<br><br>Participant outcome include, increase satisfaction and self-confidence; acquisition of knowledge, skills, and attitudes; and behaviour.<br><br>An effective simulation activity helps participants to acquire psychomotor skills; communication, teamwork, and professional behaviour; clinical reasoning, and reflective thinking<br><br>A successful simulation experience must result in improved patient safety, excellence in nursing care and reflective practice.<br><br>System outcome refers to how nurses trained with simulation contribute to saving cost (cost-effectiveness) and change in practice.	The outcome of practical skills teaching is to improve the knowledge and clinical skills of participants, improve positive patient outcomes, and system efficiency.	The outcome of practical skills teaching is to improve the knowledge and clinical skills of participants, improve positive patient outcomes, and system efficiency.	The outcome of SBCNE is focused on the participant, patient, and system. The participant outcome is substantially recorded in the literature and includes increased satisfaction and self-confidence; critical thinking; the acquisition of knowledge, skills, and attitude as well as behavioural change. The patient outcome examines how knowledge gained in simulation is used to direct patient care, resulting in positive patient outcomes and satisfaction. System outcome refers to how simulation-trained nurses contribute to cost savings (cost-effectiveness) and evidence-based practice change.
